# Conjugated Linoleic Acid and Brain Metabolism: A Possible Anti-Neuroinflammatory Role Mediated by PPARα Activation

**DOI:** 10.3389/fphar.2020.587140

**Published:** 2021-01-08

**Authors:** Elisabetta Murru, Gianfranca Carta, Claudia Manca, Valeria Sogos, Marco Pistis, Miriam Melis, Sebastiano Banni

**Affiliations:** ^1^ Department of Biomedical Sciences, University of Cagliari, Monserrato, Italy; ^2^ Neuroscience Institute, National Research Council of Italy (CNR), Cagliari, Italy

**Keywords:** conjugated linoleic acid, peroxisome proliferator-activated receptor α, brain, neuroinflammation, lipid nutrition

## Abstract

Fatty acids play a crucial role in the brain as specific receptor ligands and as precursors of bioactive metabolites. Conjugated linoleic acid (CLA), a group of positional and geometric isomers of linoleic acid (LA, 18:2 n-6) present in meat and dairy products of ruminants and synthesized endogenously in non-ruminants and humans, has been shown to possess different nutritional properties associated with health benefits. Its ability to bind to peroxisome proliferator-activated receptor (PPAR) α, a nuclear receptor key regulator of fatty acid metabolism and inflammatory responses, partly mediates these beneficial effects. CLA is incorporated and metabolized into brain tissue where induces the biosynthesis of endogenous PPARα ligands palmitoylethanolamide (PEA) and oleoylethanolamide (OEA), likely through a positive feedback mechanism where PPARα activation sustains its own cellular effects through ligand biosynthesis. In addition to PPARα, PEA and OEA may as well bind to other receptors such as TRPV1, further extending CLA own anti-neuroinflammatory actions. Future studies are needed to investigate whether dietary CLA may exert anti-inflammatory activity, particularly in the setting of neurodegenerative diseases and neuropsychiatric disorders with a neuroinflammatory basis.

## Introduction

Fatty acids (FAs) are ubiquitous biological molecules that are used as metabolic fuels, essential components of cellular membranes and regulators of signaling molecules. Any tissue has its own peculiar preferential use for FAs: some are more prone for β-oxidation (e.g., muscles), others for the formation and the processing of bioactive metabolites (e.g., brain), or FA accumulation in the form of triglycerides (e.g., adipose tissue), or for the regulation and formation of desaturated/elongated metabolites (e.g., liver) and their release into the bloodstream for their transport to different tissues. In order to properly carry out all of these metabolic pathways, some sort of intracellular “sensor” is required, which can selectively drive FA metabolism according to cell tissue requirements and upon FA tissue availability. To this aim, the “elaboration” or the processing of dietary FAs are important to fulfill tissue FA requirements. Additionally, even though within a definite range, dietary FAs affect tissue composition, thereby directly or indirectly (i.e., through their metabolites) regulating FA-derived signaling molecules.

Conjugated linoleic acid (CLA), is a group of positional and geometric isomers of linoleic acid (LA, 18:2 n-6) present in meat and dairy products of ruminants (Martins et al., 2007), and endogenously synthesized in non-ruminants and in humans (Shingfield and Wallace, 2014). The most studied isomers are c9,t11, the natural isomer mostly present in foods, and t10,c12 mainly present in nutraceutical supplements.

As a consequence, plasma basal level of c,9t11 CLA has been detected (around 15 nmoles/ml), as well as its metabolites. Interestingly, following supplementation, the plasma concentrations of c9,t11CLA and its metabolites were incorporated in a linear fashion (Mele et al., 2013).

Biological properties exerted by these isomers can be different or shared, depending on the effect and the tissue (Churruca et al., 2009) and have been shown to possess different nutritional properties (Belury, 2002; den Hartigh, 2019). However, some adverse effects have been described for the isomer c9,t11-CLA, such as increased levels of C-reactive protein (Smedman et al., 2005), a small decrease of insulin sensitivity of about 14.4 ± 16.7%, and increased levels of isoprostane, a marker of lipid peroxidation of 50 ± 40% in obese man (Riserus et al., 2004). t10,c12 CLA has also been shown to induce a small increase of isoprostane, compared with the isomer c9,t11 CLA (Tholstrup et al., 2008). However, the same group demonstrated that the isoprostane increase was not associated with risk markers of cardiovascular disease, inflammation, or fasting concentrations of insulin and glucose (Raff et al., 2008). Notably, isoprostane increase is suggested to not be ascribed to an ongoing lipid peroxidation, but rather to its reduced catabolism in peroxisomes due to a competition with CLA (Iannone et al., 2009). In order to evaluate potential adverse effects of CLA in humans, Wanders et al. designed a study on 61 healthy volunteers, which were administered with 20 g/days of a c9,t11 and t10,c12 CLA isomers 80/20 mixture (Wanders et al., 2010a), and showed no changes in either lipoprotein profile or in liver and kidney function (Wanders et al., 2010b).

The numerous and contrasting biological effects reported for CLA (Benjamin et al., 2015) are probably due to its pleiotropic properties, and may not be explained by a single biochemical mechanism (Kennedy et al., 2008), although they are generally ascribed to its activities on lipid and energy metabolism. CLA metabolism, extensively studied especially in rodents (Banni et al., 1995; Belury and KempaSteczko, 1997; Sebedio et al., 1997), probably influences the metabolism of n-6 polyunsaturated FAs (PUFA) by competing for their formation (Banni et al., 1999; Banni, 2002) and enhances the formation of docosahexaenoic acid (DHA, 22:6 n-3) in experimental animals (Piras et al., 2015; Carta et al., 2019) and humans (Murru et al., 2018), by inducing peroxisomal β-oxidation (Ferdinandusse et al., 2003).

In a study performed on neonatal piglets fed with or without supplementation of CLA for 16°days, aimed at investigating the incorporation and metabolism of CLA isomers in brain tissue (Lin et al., 2011), the authors showed that CLA significantly affected the biosynthesis of long-chain PUFA in liver and brain tissues by inhibiting LA elongation/desaturation pathways. The inhibitory effects were dependent on the reduced activity of delta 6 desaturase/elongase-5 (Δ6D/Elovl-5) and especially the alternate Elovl-A elongation/desaturation pathway and were associated with the fat content and the corresponding PUFA levels in the dietary fat (Lin et al., 2011).

In addition, it has been recently shown that dietary intake of CLA induced the biosynthesis of oleoylethanolamide (OEA) and palmitoylethanolamide (PEA) in the liver of obese Zucker rats, an effect associated to a reduced hepatic lipid deposition (Piras et al., 2015). OEA and PEA are natural ethanolamides of oleic acid (OA, 18:1 n-9) and palmitic acid (PA, 16:0), respectively. OEA reduces food intake and body weight gain in obese rats (Fu et al., 2005), stimulates lipolysis and FA oxidation (Guzman et al., 2004), reduces the content of triacylglycerol (TAG) in both the liver and adipose tissue (Guzman et al., 2004). All of these properties may be attributed to CLA ability to activate specific receptors such as peroxisome proliferator-activated receptors (PPAR) α, β/δ and AMP-activated protein kinase (AMPK) (Trinchese et al., 2020). Despite the high brain expression of PPARβ and its possible role in regulating the metabolism of FAs and/or cholesterol, in inflammation processes and antioxidant mechanisms brain (Hall et al., 2008), very little is known nowadays about how CLA regulates this nuclear receptor in the healthy and diseased brain.

PPARα is a ubiquitous ligand-activated transcriptional factor that belongs to the family of nuclear receptors. PPARα regulates the expression of genes involved in FA metabolism, β-oxidation in both mitochondria and peroxisomes, acting as an intracellular FA sensor and regulating FA trafficking according to cell tissue requirements upon tissue FA availability. The discovery that FA are endogenous ligands of PPARα occurred when Gottlicher et al. (Gottlicher et al., 1992) tested the capability of FA to activate PPARα upon the observation that the class of synthetic PPARα agonists fibrates and FA displayed similar biological activity. Indeed, fibrates and FA induce a conformational change of the PPARs, triggering the transcription of genes encoding for metabolic and cellular processes such as FA β-oxidation and adipogenesis representing key mediators of lipid homeostasis (Echeverria et al., 2016). Along with other studies, a bewildering array of compounds activating PPARα has been discovered (Schoonjans et al., 1996). However, all the efforts made to demonstrate that these compounds directly bind to PPARα have failed. This has led to the hypothesis that these compounds alter FA metabolism, which indirectly leads to the accumulation of endogenous PPARα ligands (Gottlicher et al., 1993). A ligand-binding assay has been developed that facilitates the identification of ligands for PPARα and PPARβ/δ. It has been found that fibrates and specific FA/eicosanoids can bind to these receptors. This indicates that FA simultaneously serve as intermediary metabolites and as primary regulators of transcriptional networks (Forman et al., 1997).

Even though the comparison of the ligand activity among different fatty acids is extremely difficult to assess because their cellular concentration may vary greatly in different tissues, Krey et al. (1997) using co-activator-dependent receptor ligand assay (CARLA), screened several FA and found LA and linolenic acids the most affine to PPARα. Interestingly, Moya-Camarena et al. found that CLA was by far more potent inducer than LA (Moya-Camarena et al., 1999).

PPARα is also a key regulator of inflammatory responses (Delerive et al., 2001; Korbecki et al., 2019): its anti-inflammatory effects are primarily mediated through their abilities (shared with the other PPARs) to trans-repress (Ricote and Glass, 2007) the functions of many activated transcription factors, such as the transcription factor nuclear factor-κB (NF-κB), signal transducers and activators of transcription (STATs), activator protein 1 (AP1) and nuclear factor of activated T cells (NFAT) (Daynes and Jones, 2002; Wahli and Michalik, 2012). Data suggested that these metabolic and anti-inflammatory effects are not restricted to the periphery but also occur in the CNS. Therefore, if CLA is an avid ligand of PPARα, it may as well possess anti-neuroinflammatory properties. If so, it must be first incorporated into cell tissue lipids in order to bind to PPARα.

### Conjugated Linoleic Acid Incorporation and Metabolism in Brain Tissue in Rats and Humans

In peripheral tissues, CLA incorporation is prompt and its deposition occurs particularly in neutral lipids (NL). CLA and its desaturated and elongated metabolites are likely biosynthesized and then transported to extrahepatic tissues, as evidenced by their high concentration also in plasma and adipose tissue after dietary CLA administration. Given that modification of FA profile in the brain by dietary FAs is quite difficult (Zamberletti et al., 2017; Carta et al., 2019; Carta et al., 2020), this is also true for dietary CLA.

In rats fed a diet supplemented with 150 mg/day of 9c,11t or 9t,11t or 10t,12c or 10t,12t isomers for six days, only minor changes in CLA brain concentrations were found (Alasnier et al., 2002). This might be due to either 1) a preferential incorporation of CLA in tissue TAG (Banni et al., 2001; Paterson et al., 2002) and the relative virtual lack of this lipid fraction in brain tissue; or 2) a very selective and steady incorporation of FAs in the brain (Salem and Niebylski, 1995); or else 3) a rapid CLA metabolism to other conjugated FAs in the brain.

Fa and co-workers (Fa et al., 2005) administered a single dose of CLA (2 g by gavage) to female Sprague–Dawley rats to monitor its incorporation and metabolization up to 24 h. Confirming previous research, CLA incorporation was much lower in the brain than in the other tissues examined. At 24 h CLA isomer concentrations were both increased by four folds in plasma and liver and two folds in brain, whereas in adipose tissue 9c,11t isomer increased six folds and t10,c12 by four folds. However, a relative high accumulation of CLA metabolites was found, particularly products of peroxisomal β-oxidation related to the content of the precursor. The discrete levels of the two CLA isomers measured in plasma could be ascribed to the different rates of hydrolyzation of the isomers in chylomicron TAG by lipoprotein lipase. In the brain, the level of the t10,c12 isomer was lower than that of the c9,t11 isomer, probably because of the enhanced metabolism of t10,c12 with respect to c9,t11, as shown by higher concentrations of t10,c12 metabolites. t10,c12 seemed to be β-oxidized very efficiently in all tissues, particularly in the brain. Products of peroxisomal β-oxidation of CLA were detected in experiments *in vivo* and *in vitro,* confirming that CLA could act as a ligand to brain PPARα (Cullingford et al., 1998; Moreno et al., 2004).

Interestingly, astrocytes may play a crucial role on CLA metabolization as confirmed by *in vitro* studies (Fa et al., 2005). Cerebellar astrocytes were isolated from 7 days-old Sprague–Dawley rats and treated with 100 µM of CLA mixture, were shown to produce relevant concentration of CLA metabolites. These results suggest that activation of PPAR-mediated differentiation pathways could be a mechanism by which CLA could exert beneficial effects on the brain, especially in disorders characterized by an impairment of peroxisomal β-oxidation inducing demyelination of nerve fibers. This is the case of X−linked adrenoleukodystrophy (ALD), characterized by an abnormal accumulation of very long-chain FAs (VLCFA), owing to defects of the ALDP, an integral peroxisomal membrane protein (Douar et al., 1994), specifically 26:0 which is almost exclusively β-oxidized in peroxisomes. The increase of VLCFA intercalated in the membrane may account for demyelination and increased immunoreactivity (Baes and Aubourg, 2009). A mixture of glycerol trioleate and glycerol trierucate, 4:1, Lorenzo’s oil (LO), reduced plasma levels of VLCFA competitively inhibiting the elongase that forms VLCFA. However, LO decreased plasma VLCFAs, while it did not prevent ALD (Di Biase and Markus, 1998), probably because erucic acid (22:1) may not accumulate sufficiently into brain lipids due to very low desorption (Poulos et al., 1994; Hamilton et al., 2007). Cappa and co-workers (Cappa et al., 2012) treated five ALD women, who were not treated with LO therapy at the study entry, with a mixture of LO (40 g/day) plus a mixture of CLA (5 g/day) for 2 months, a dose sufficiently high to be incorporated in the central nervous system (CNS). Treatment with the mixture LO + CLA significantly increased CLA levels to 50 nmol and 1,2 nmol/ml (around five and two folds vs. the baseline levels) in plasma and CSF respectively. This approach was based on the hypothesis that CLA may act synergistically with LO, as CLA is a high-affinity ligand of PPARα (Moya-Camarena et al., 1999), and might thereby induce the key enzymes for peroxisomal β-oxidation (Reddy and Hashimoto, 2001).

CLA may also contribute to the shortening of 24:0 and ameliorate eicosanoid and oxidative stress product catabolism by increasing peroxisomal β-oxidation, acting as an anti-inflammatory and antioxidative factor. In all patients, the LO + CLA mixture treatment decreased plasma levels of VLCFA, while increased CLA levels in plasma and cerebrospinal fluid (CSF) and the docosahexaenoic acid/eicosapentaenoic acid (22:6/20:5) ratio, an indirect marker of peroxisomal β-oxidation induced by PPARα (Ferdinandusse et al., 2002). Cappa et al. study demonstrated for the first time that CLA promptly crosses the human blood-brain barrier (Cappa et al., 2012). Furthermore, because there was a correlation between changes of CLA concentrations in CSF and plasma, this may suggest that the linear dose-response found in plasma of experimental animals (Banni et al., 1999) and more recently in humans (Murru et al., 2018) may also occur in CSF.

### *In Vivo* Brain CLA Effects in Experimental Animals and Humans

Although CLA isomers are incorporated and metabolized in the brain of several species, pieces of evidence about its impact on brain function are still limited, not being adequately addressed in both experimental animals and humans.

Different studies evaluated the potential benefits of CLA on neurodegenerative diseases as Alzheimer’s disease (AD) or other disorders that may contribute to dementia in elderly people by modulating the neuroinflammation in the brain. The brain seems to be exceptionally susceptible to peroxidation, and neurodegenerative diseases are accompanied by the activation of defensive mechanisms such as astrogliosis (Rojo et al., 2014), macroautophagy (Filomeni et al., 2015) and the activation of nuclear factor-E2-related factor 2 (Nrf2), by controlling cell redox homeostasis through the production/recycling of the intracellular antioxidant glutathione (Calabrese et al., 2010; Johnson and Johnson, 2015).

Previous studies indicated that CLA improved systemic antioxidant and detoxifying defences via the activation of the Nrf2 pathway (Mollica et al., 2014; Bergamo et al., 2016). It is possible to hypothesize that its dietary supplementation might alleviate age-dependent neurodegenerative signs as showed in neuropsychiatric lupus old/diseased MRL/MpJ-Faslpr mice, considered an animal model of depression because of the spontaneous development of depressive-like behavior and autoimmune/oxidative stress signs (Monaco et al., 2018; Cigliano et al., 2019). In this study, mice (20- to 22-weeks-old) fed for five weeks with a daily supplementation of synthetic CLA mixture displayed a reduction of all pathological features in the brain when compared to young mice or healthy controls. This finding indicates a preventive effect of CLA against age-associated neuronal injury and hyper-activation of oxidative stress-activated compensatory mechanisms (Monaco et al., 2018).

An altered phospholipid (PL) metabolism may be associated with the loss of synapses and neurons, the formation of senile plaques and neurofibrillary tangles in AD (Pettegrew, 1989; Farooqui and Horrocks, 1994) and the decreased membrane fluidity. All these factors may be associated with functional and degenerative changes in the brain (Erin et al., 1986). One of the CLA functions is to alter, mostly decreasing, prostaglandin formation in a tissue-specific manner (Whigham et al., 2001; Belury, 2002; Whigham et al., 2002). Arachidonic acid (ARA, 20:4 n-6), the principal FA esterified in sn-2 position of PL, can be specifically cleaved by phospholipases A2 (PLA2) (Dennis, 1987; Glaser et al., 1990; Wightman and Dallob, 1990; Farooqui et al., 2000) and converted into its inflammatory metabolite, prostaglandins G2 (PGE2), by cyclooxygenases (COX) enzyme (Miyamoto et al., 1976; Van der Ouderaa et al., 1977; Pagels et al., 1983; Tanaka et al., 1987; Ujihara et al., 1988). However, in the brain, ARA is mainly reincorporated into PLs (Rapoport, 1999; Leslie, 2004), through which can modulate neuronal function by various mechanisms (Katsuki and Okuda, 1995; Farooqui et al., 1997). Thus, regulation of PLA2 activity is important to maintain basal levels of ARA, lysophospholipids and to perform normal brain function (Farooqui et al., 2000; Phillis and O'Regan, 2004). The reduction of PLA2 activity in the brain may be involved in neuronal degeneration (Gattaz et al., 1995; Ross et al., 1998; Schaeffer et al., 2010). In cholinergic neurons, for instance, PLA2 controls the breakdown of phosphatidylcholine to produce choline for acetylcholine synthesis, and may contribute to the cholinergic deficit observed in AD (Blusztajn and Wurtman, 1983; Blusztajn et al., 1987). To date, a few studies concerning the effects of CLA on the activity and expression of PLA2 in *in vivo* tissues, especially in the brain, are available (Akdis et al., 2000; Eder et al., 2003; Ringseis et al., 2006; Stachowska et al., 2007). In the hippocampus of Wistar rats fed with a diet high in CLA, the mRNA levels of *pla2* were increased together with the augmented enzymatic activity of PLA2 enzyme, and a potential correlation with memory improvement was observed (Gama et al., 2015).

These discrepant results in the literature suggest the importance of more studies aimed at a precise explanation of the relationship between PL metabolism and cognition. Animal models and clinical studies suggested that the activity and gene expression of PLA2 may involve the activation of PPARs (Kummer and Heneka, 2008). In fact, while especially PPARγ has been implicated in neural cell differentiation and death, as well as in inflammation and neurodegeneration in astrocytes (Combs et al., 2000; Heneka et al., 2000; Daynes and Jones, 2002; Xu and Drew, 2007; Bernardo and Minghetti, 2008), PPARα is involved in acetylcholine metabolism (Farioli-Vecchioli et al., 2001) and is related to excitatory amino acid neurotransmission and oxidative stress defence (Moreno et al., 2004). Interestingly, Sergeeva et al. showed that the expression of PLA2 was inhibited by PPARα and PPARγ agonists in naive astrocytes, but was increased by PPARγ activation in lipopolysaccharide (LPS)-stimulated astrocytes (Sergeeva et al., 2010). Thus, CLA-induced enhancement of PLA2 gene expression may be mediated by the activation of PPARγ in the brain and might depend on the inflammatory status of the tissue (Gama et al., 2015).

There are few studies examining the mechanisms of CLA modulation of eicosanoids in the brain (Nakanishi et al., 2003). A reduction of ARA-derived eicosanoids by CLA is explained by the inhibition of the level of mRNA, protein, or activity of the COX-1 constitutive enzyme and/or the COX-2 inducible form (Bulgarella et al., 2001). COX-2 mRNA is elevated in the brain (Kaufmann et al., 1996; Yasojima et al., 1999) and is associated with inflammatory responses induced by stimuli including cytokines, tumor promoters, and growth factors (Seibert et al., 1995; Cao et al., 1996). Notably, CLA supplementation in maternal diet during pregnancy significantly reduced PGE2 levels in the cerebrum of mice at weaning, an effect that seems to persist until adulthood. CLA probably mitigates the toxic impact of β-amyloid in neurons by decreasing amyloid precursor protein gene expression and its holoprotein synthesis (Mattson et al., 1992; Lee et al., 1999; Melchor et al., 2000).

In female X-linked adrenoleukodystrophy patients, CLA exerted anti-neuroinflammatory activity (Cappa et al., 2012). In fact, changes in FA profile, especially in CLA incorporation, resulted in improved somatosensory evoked potentials and reduced IL-6 levels in CSF (Cappa et al., 2012). In addition, CLA crosses the human placenta to the fetus (Martin et al., 2000; Elias and Innis, 2001), is present in small amounts in human milk (McGuire et al., 1997), and is incorporated into infant plasma lipids (Innis and King, 1999). In the progeny of rat dams fed with goat milk containing CLA, the anxiety-like behavior was reduced, physical growth ameliorated and cortical electrical activity improved, demonstrating the importance of CLA on neonatal development and health (Soares et al., 2013). Thus, CLA may favor the neurodevelopment occurring during the embryonic phase and the initial phases of life (Muller et al., 2015). Queiroz et al. reported that maternal supplementation with different CLA concentrations (1% and 3%) during gestation and lactation in rats positively affected neurodevelopment by anticipating reflex maturation and improving working memory in the offspring. These effects might be indirect or through some metabolites since CLA was found in the brain only in trace amounts (Queiroz et al., 2019).

CLA can also exert beneficial effects on fat deposition and body weight and might facilitate decreased food intake and increased energy expenditure (Wang and Jones, 2004; Salas-Salvado et al., 2006). To better elucidate the mechanism of the actions of exogenous CLA administration on the expression of hypothalamic neuropeptides known to regulate food intake, a group of researchers demonstrated that direct intracerebroventricular administration of CLA in rats inhibited appetite regulation, which was related with the decreased expression of the orexigenic neuropeptides Y (NPY) and agouti-related protein (AgRP) (Cao et al., 2007). Remarkably, PPARα activation has been shown to produce satiety and reduces body weight gain in wild-type mice, but not in mice deficient in PPARα (Fu et al., 2003).

### Effects of Conjugated Linoleic Acid on Brain Cells *in vitro*

Molecular mechanisms underlying the effects of CLA in the CNS were studied in different neural cell culture models. Astrocytes represent the most abundant type of glial cells and are responsible for a large variety of functions in the healthy CNS, including synaptogenesis, neuronal transmission and synaptic plasticity. Astrocytes also participate in immune and inflammatory responses and produce a wide range of factors, such as cytokines or chemokines that contribute to the inflammatory state of the CNS after injury or during neurodegenerative diseases (Colombo and Farina, 2016).

CLA induces a decrease in inflammatory factors in primary human astrocyte culture, suggesting a potential nutritional role in modulating astrocyte inflammatory response. Both c9,t11 and t10,c12 isomers determine a downregulation of proinflammatory cytokine expression, such as tumor necrosis factor-α (TNF-α), interleukin-1β (IL-1β), and RANTES (regulated upon activation, normal T cell expressed and secreted), but only t10,c12 decreases ARA production. Interestingly, CLA exerts anti-inflammatory activity in astrocytes modifying FA metabolism as suggested by the increase of the 22:6/20:5 ratio (Saba et al., 2019), indicating an increased peroxisomal β-oxidation induced by PPARα.

In AD, amyloid precursor protein (APP) cleavage by β-secretase (BACE1) generates β-amyloid peptide (Aβ) that accumulates and form neurotoxic plaques outside the cells. Alternative processing of APP by α-secretase generates soluble APPα that has neurotrophic and neuroprotective properties. Calpain is a Ca2+-dependent protease which activity is dysregulated in AD, causing an increase of BACE1 expression, tau phosphorylation, oxidative stress and other excitotoxicity assaults (Mahaman et al., 2019).

CLA has been proposed as an adjuvant for the treatment and the prevention of AD since it may control the abnormal processing of APP. In the human neuroblastoma cell line SH-SY5Y, CLA induces a decrease of BACE1 expression and an increase of the extracellular secretion of soluble APPα but does not affect the levels of APP. These effects of CLA are mediated by PPARγ activation (Li et al., 2011). Moreover, CLA acts as a potent and selective µ-calpain inhibitor as reported by Lee and collaborators (Lee et al., 2013) that showed a neuroprotective effect of CLA against Aβ and ROS-induced toxicity in SH-SY5Y cells. Moreover, CLA decreased the levels of proapoptotic proteins and tau phosphorylation and was able to prevent Aβ oligomerization and fibrillation.

CLA exerts a neuroprotective effect even in glutamate excitotoxicity in primary culture of rodent cortical neurons. Joo and Park showed inhibition of glutamate- and NMDA-induced cell death by a high concentration of CLA (500 µM) in cultured rat cortical neurons (Joo and Park, 2003). On the other hand, Hunt et al. more recently observed similar effects, but at CLA concentration likely achieved by dietary supplementation. In fact, 30 µM c9,t11 protects mouse cortical neurons from glutamate-induced excitotoxic death and increases levels of the anti-apoptotic BCL-2 protein, while t10,c12 isomer has no significant effect (Hunt et al., 2010).

Neural stem cells (NSC) and neural precursor cells (NPC) are self-renewing, multipotent cells that give rise to neurons and glial cells during development of the CNS, but continuously generate functional neurons in specific brain regions throughout life. c9,t11 promotes proliferation in neurospheres derived from rat NPC and increases cyclin D1 expression, while the isomer t10,c12 had the opposite effect (Wang et al., 2011). Moreover, treatment with c9,t11 promotes neuronal differentiation of rat NSC, increasing Tuj-1-positive cells. This effect is due in part to the increase of the bHLH transcription factor HES6 expression (Okui et al., 2011).

Several data suggest that also in the brain some of the effects exerted by CLA can be ascribed to its activation of PPARα. In fact, we have previously shown that PPARα activation by synthetic agonists increased PEA and OEA biosynthesis, as we also showed in liver and muscle *in vivo* (Melis et al., 2013a; Trinchese et al., 2018; Trinchese et al., 2020).

**FIGURE 1:**
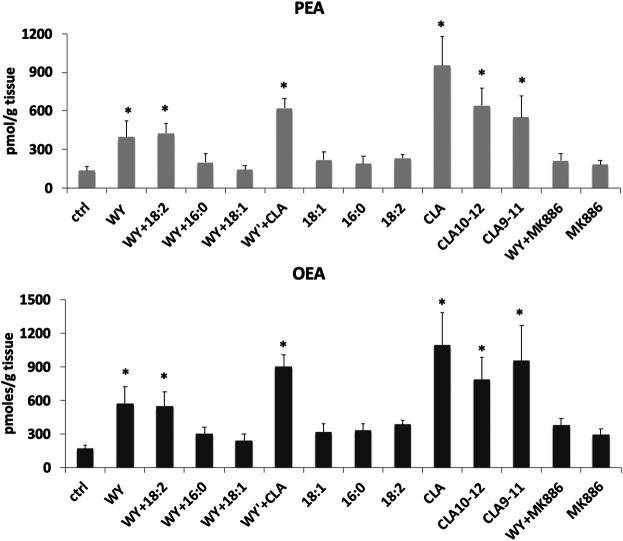
Concentrations of palmitoylethanolamide (PEA) and oleoylethanolamide (OEA) analyzed by LC-MS as described in (Piras et al., 2015), in rat horizontal slices containing the midbrain incubated for 1 h with 100 µM of synthetic and endogenous ligands of PPARα or their vehicle: agonist WY14643 (WY), oleic acid (18:1), palmitic acid (16:0), linoleic acid (18:2), c9-t11, t10-c12, and a mixture of both CLA isomers (CLA), or PPARα antagonist (MK886). Error bars represent SD; *n* = 6. * denote significant differences (*p* < 0.05), vs. control (one-way ANOVA).

**FIGURE 2:**
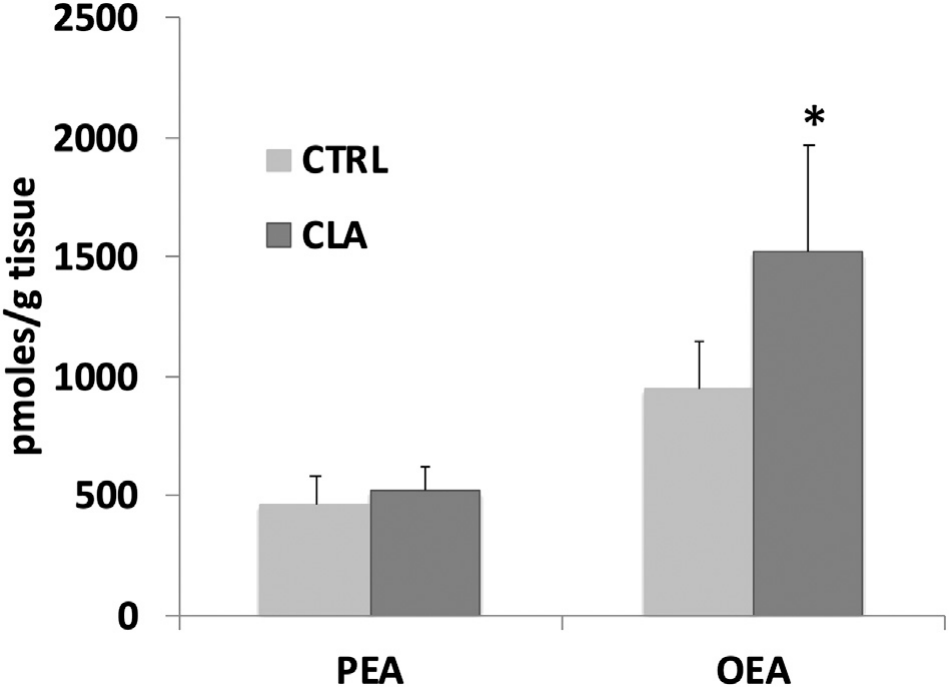
Palmitoylethanolamide (PEA) and oleoylethanolamide (OEA) levels analyzed by LC-MS as described in (Piras et al., 2015), in the hypothalamus of gavage-treated mice, fed a standard diet, with a single dose (90 µg/10 g of body weight) of CLA (CLA) or olive oil (Ctrl). *N* = 6. Error bars represent SEM; * denote significant differences (*p* < 0.05) vs. control (one-way ANOVA).

In unpublished experiments, we evaluated the impact of CLA isomers or the mixture of both isomers, compared to synthetic (WY14643) and endogenous (FA) ligands or antagonist (MK886) of PPARα, on FA metabolism and biosynthesis of endogenous PPARα ligands OEA and PEA in midbrain slices. Our unpublished data showed that CLA is able to increase OEA and PEA levels in rat midbrain slices incubated for 60 min with 100 µM of CLA mixture or pure c9,t11 and t10c12 isomers (Figure 1). In addition, gavage-treated mice, with a single dose (90 µg/10 g of body weight) of CLA or olive oil (Ctrl), fed a standard diet, similarly showed an increase of OEA levels in the hypothalamus (Figure 2).

Thus, our data strongly suggest that CLA exerts its activity, at least in part, via PPARα in brain tissues similarly to peripheral tissues. What are the potential implications of CLA activation of PPARα in the brain? Does it play a synergistic role with those exerted in peripheral tissues or may have further potential benefits? This point is quite relevant, given the pleiotropic effects of PPARα activation in the brain.

### Effect of Peroxisome Proliferator Activated Receptor Alpha in Brain

PPARα displays a specific pattern of expression in the CNS, with higher levels in thalamic, mesencephalic and cranial motor nuclei, the reticular formation and the large motoneurons of the spinal cord and lower levels in the amygdala, prefrontal cortex, nucleus accumbens, ventral tegmental area and substantia nigra *pars compacta* (Moreno et al., 2004; Fidaleo et al., 2014; Warden et al., 2016). PPARα is also expressed by ependymal and astroglial cells, but not by oligodendrocytes (Moreno et al., 2004). Interest in the role of PPARα in the CNS has been fueled by the evidence that these nuclear receptors regulate a wide range of physiological functions in neuronal and glial cells, and might play a role in higher brain functions including memory consolidation and modulation of pain perception (Fidaleo et al., 2014). In this regard, it is noteworthy that the neuronal effects of PPARα agonists cannot only be explained through transcriptional effects canonically ascribed to PPARα activation but also via rapid non-genomic mechanisms (Melis and Pistis, 2014; Pistis and Muntoni, 2017). Unlike genomic effects, which occur with a time lag of minutes to hours and days, these events take place over a very rapid time frame (i.e., seconds to a few minutes). This timescale is considered too rapid to be attributed to the biosynthesis of mRNA or proteins and is often unaffected by inhibitors of transcription or translation. Solid evidence that PPARα exerts rapid non-genomic effects also derives from the platelets, anucleate cells, where the PPARα ligands fibrates display antiaggregant effects by binding to and repressing PKCα, increasing intracellular levels of cAMP levels (Ali et al., 2009) and inhibiting ADP-stimulated platelet activation (Unsworth et al., 2018).

In the brain, non-genomic actions have been described in the cross-talk between PPARα and nicotinic acetylcholine receptors (nAChRs) (Melis and Pistis, 2014). In midbrain dopamine neurons, α7nAChRs-induced Ca^2+^ influx triggers the synthesis of endogenous PPARα ligands which, in turn, activate PPARα to induce phosphorylation of β2 subunits of α4β2* nAChRs (Melis et al., 2010; Melis et al., 2013b). This interaction is hypothesized to serve as negative feedback to fine-tune the activity of nicotinic cholinergic transmission in the dopamine system, as activation of low-affinity α7nAChRs by an excessive cholinergic tone negatively regulates either number and/or function of high affinity β2*nAChRs. Consistently, a PPARα antagonist prevents the inhibitory effects of an α7nAChR agonist on nicotine reward in a mouse conditioned place preference paradigm, suggesting that α7nAChR activation attenuates nicotine place preference via a PPARα-dependent mechanism (Jackson et al., 2017). Accordingly, an α7nAChR agonist prevents nicotine stimulating effects on spontaneous locomotor activity in mice, a condition in which the activation of β2*nAChRs expressed on dopamine cells is necessary (Picciotto et al., 1998) and of PPARα sufficient (Melis and Pistis, unpublished data). Dysregulation of dopamine-acetylcholine interplay occurring in pathological conditions such as stress, drug addiction, schizophrenia and depression, might benefit from PPARα activation (Melis and Pistis, 2014). Such PPARα-acetylcholine interaction also takes place in other brain areas receiving strong impact of cholinergic inputs such as the sensorimotor cortex (Puligheddu et al., 2013; Puligheddu et al., 2017). These findings provided the rationale for using PPARα ligands, i.e., the clinically approved fibrates, as add-on therapy in neurological disorders caused by a gain of function of nAChRs, such as in sleep-related hypermotor epilepsy (SHE, previously named nocturnal frontal lobe epilepsy, NFLE). Thus, the powerful actions exerted by PPARα via dual genomic and non-genomic mechanisms might contribute to strengthening the rationale for these nuclear receptors as a promising therapeutic target in the CNS, especially when considering that neuroinflammation appears to be involved in the pathophysiology of diverse psychiatric and neurological illnesses (Pistis and Muntoni, 2017; Tufano and Pinna, 2020). In particular, mounting evidence points to a relationship between neuroimmune function and neurodevelopment disorders such as autism and schizophrenia (Martínez-Gras et al., 2011; Chase et al., 2015; Gottfried and Bambini-Junior, 2018; Müller, 2018) as well as mood disorders (Scheggi et al., 2016; Pfau et al., 2018; Tufano and Pinna, 2020). In addition, it has been shown that fenofibrate reduces neuroinflammation, and blocks neurodegeneration *in vivo* (Esmaeili et al., 2016).

## Conclusion

Intriguingly, while possible beneficial effects of CLA in peripheral tissues, probably mediated by PPARα activation, have been the object of several studies in experimental animals (Trinchese et al., 2020), and humans (Murru et al., 2018), a few studies on possible positive actions in brain function and neuroinflammation through PPARα activation are available. Our findings that CLA increases PEA and OEA levels in peripheral tissues (Piras et al., 2015) and in mouse brain (Figure 2), and that this increase occurs *in situ* in the brain, similarly to PPARα agonists [Figure 1 and (Melis et al., 2013a)], supports the hypothesis that PPARα activation induces PEA and OEA biosynthesis thereby sustaining PPARα activation with a positive feedback mechanism.

Notably, CLA may indirectly, via sustaining OEA and PEA biosynthesis, activate receptors other than PPARα, like GPR119 and TRPV1, which are also implicated in metabolism regulation and anti-inflammatory activity, respectively (Godlewski et al., 2009; Ambrosino et al., 2013). Accordingly, increased dairy product intake is associated with improved cognitive function in humans (Crichton et al., 2012; Park and Fulgoni, 2013), whether these effects may be ascribed to CLA and/or other components in dairy products has not been elucidated yet. Thus, future studies should be devoted to investigating whether dietary CLA may positively modify brain metabolism, though PPARα activation, and thereby exert anti-inflammatory activity, particularly in the setting of neuropsychiatric disorders with neuroinflammatory bases.

## Author Contributions

EM, GC, and SB, conceived the topic of the review and organised the ms structure. CM, EM, GC, MM, MP, and SB performed and designed the experiments described in the unpublished data. VS reviewed the manuscript and contributed to the draft in particular on the in vitro literature. SB supervised the manuscript draft. All Authors contributed to the discussion and review and editing of the manuscript.

## Conflict of Interest

The authors declare that the research was conducted in the absence of any commercial or financial relationships that could be construed as a potential conflict of interest.
